# Chemical engineering of nucleic acid nanodevices: From molecular modification to biomedical applications

**DOI:** 10.1016/j.omtn.2026.103107

**Published:** 2026-10-08

**Authors:** Fang He

**Affiliations:** 1 State Key Laboratory of Chemo and Biosensing, College of Chemistry and Chemical Engineering, Hunan Provincial Key Laboratory of Biomacromolecular Chemical Biology, Hunan University, Changsha, China

## Main text

Nucleic acids have emerged as versatile molecular building blocks for biomedical technologies owing to their programmability, molecular recognition capabilities, and predictable self-assembly properties. Advances in DNA and RNA nanotechnology have facilitated the development of diverse nucleic acid nanodevices, including molecular sensors, therapeutic delivery systems, logic circuits, and responsive nanomachines.^1^ However, despite their unique design potential, the translation of nucleic acid nanodevices into biomedical applications remains challenging due to their limited stability, rapid degradation in biological environments, and insufficient spatiotemporal control over their behaviors and functions *in vivo*.^2^

In our recent review published in *Chemical Communications*,^3^ we systematically summarized how chemical engineering strategies improve the stability, controllability, and biocompatibility of nucleic acid nanodevices (Figure 1). Here, chemical engineering encompasses molecular modification, structural engineering, and programmable control of biological functions. Beyond improving nuclease resistance and molecular stability, these approaches enable regulation of the structural properties and therapeutic functions of nucleic acid-based systems. Through hierarchical engineering at the molecular and structural levels, chemically engineered nucleic acid nanodevices can sense biological signals, process molecular information, and execute therapeutic functions.

**Figure 1:**
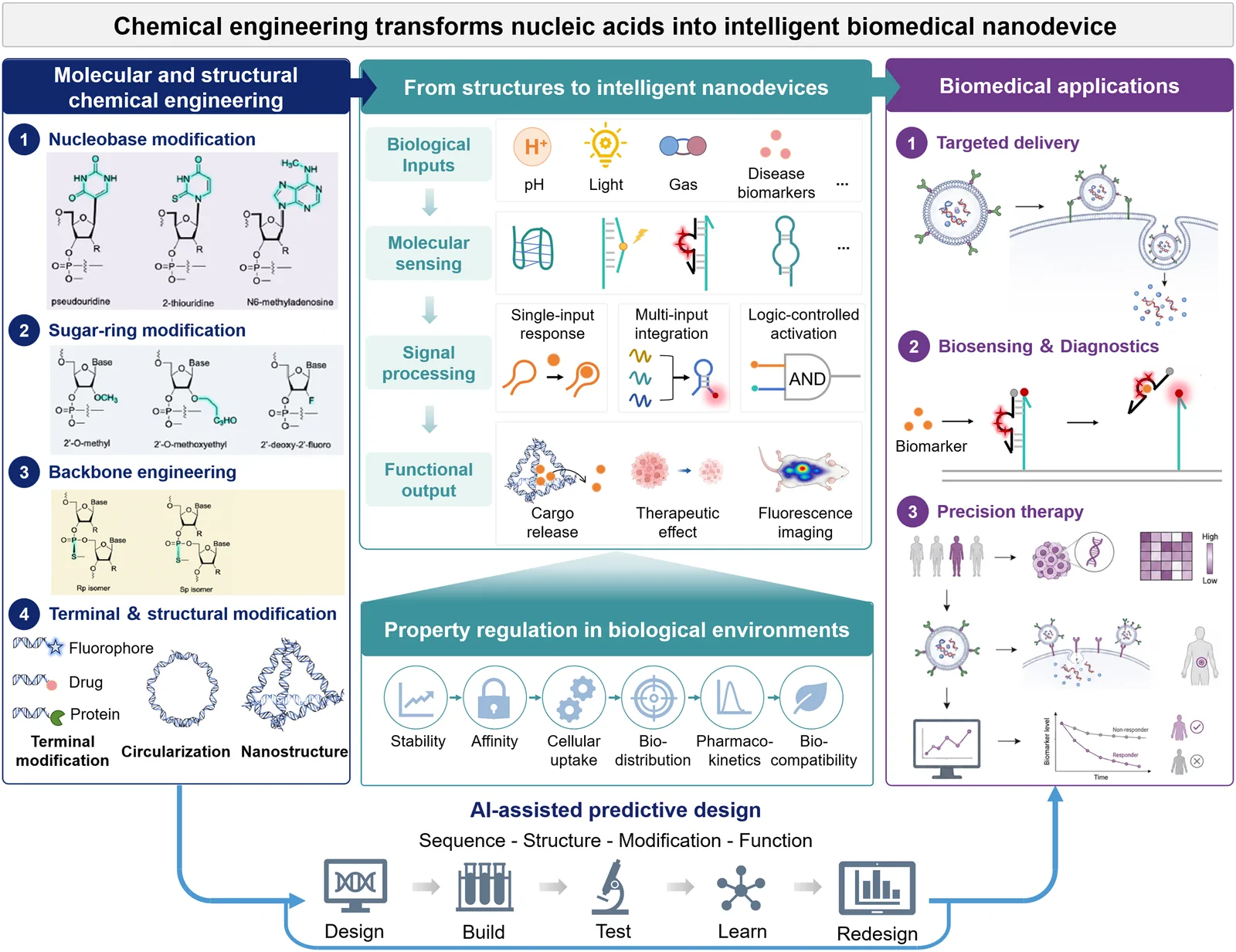
Chemical engineering transforms nucleic acids into intelligent biomedical nanodevices Molecular and structural engineering, including nucleobase, sugar, backbone, terminal, and higher-order structural modifications, provides a versatile toolbox for regulating the stability, affinity, cellular uptake, biodistribution, pharmacokinetics, and biocompatibility of nucleic acid nanodevices. These engineered properties enable biological signal sensing, molecular information processing, and programmable functional outputs through single-input responses, multi-input integration, and logic-controlled activation. AI-assisted predictive design further integrates sequence, structure, modification, and function through iterative design-build-test-learn cycles, facilitating the development of nucleic acid nanodevices for targeted delivery, biosensing and diagnostics, precision therapy, and theranostic applications.

At the molecular level, chemical modifications regulate the stability, conformation, and recognition affinity of nucleic acid nanodevices. Nucleobase modifications can preserve and even enhance Watson-Crick base pairing and base stacking while introducing additional fluorophores or functional groups for molecular recognition. Modifications of the phosphate backbone, such as phosphorothioate substitution, improve resistance to enzymatic degradation and prolong biological persistence. Sugar-ring modifications, including 2′-O-methyl, 2′-fluoro, and locked nucleic acid (LNA) modifications, further regulate duplex stability, binding affinity, conformational rigidity, and pharmacokinetic properties.^4^ Chemical engineering can also be implemented at the termini, interfaces, and higher-order structural levels of nucleic acid assemblies. Terminal functionalization and ligand conjugation enable the incorporation of targeting moieties, fluorophores, drug, protein, and other functional components. Meanwhile, polymer conjugation and circularization strategies can modulate cellular uptake and biodistribution, while enhancing resistance to enzymatic degradation and improving structural stability.^5^ Together, nucleobases, backbones, sugar rings, and terminal/structural modifications have defined a highly tunable chemical space that not only enhances biostability, delivery, and pharmacokinetics but also provides the essential molecular foundation for constructing nanodevices capable of stimulus-responsive behavior.

Beyond improving stability and delivery, chemical engineering enables nucleic acid nanodevices to respond to biological or externally applied signals. Many early nucleic acid nanostructures were designed primarily as predefined molecular architectures, whereas emerging systems increasingly incorporate chemical switches that convert environmental signals into structural rearrangements or functional outputs. Representative examples include pH-, light-, and gas-responsive nucleic acid systems.^6^ pH-responsive devices exploit acidic tumor microenvironments or intracellular compartments to trigger conformational transitions and cargo release. Light-responsive systems use photoactive chemical groups to achieve externally controlled activation with high spatial and temporal precision. Small molecule- and gas-responsive strategies further extend the sensing repertoire of nucleic acid nanodevices by using endogenous chemical signals as molecular inputs. These approaches demonstrate how chemical engineering can expand the functional roles of nucleic acids beyond structural scaffolds and enable molecular systems capable of sensing and responding to complex biological conditions. Building on these single-input responsive mechanisms, multi-input and logic-controlled systems enable more sophisticated molecular decision-making. By integrating multiple recognition, processing, and actuation modules, multi-input and logic-controlled nucleic acid nanodevices can process complex biological information, distinguish disease-associated signal combinations, and initiate functional outputs only when predefined molecular conditions are satisfied.^7^ Such designs represent a shift in nucleic acid nanotechnology from structural construction toward molecular information processing.

This increasing design complexity highlights the need for rational engineering approaches. The performance of nucleic acid nanodevices is governed by multidimensional relationships among sequence, secondary structure, chemical modification, assembly topology, and biological environment. Conventional trial-and-error approaches are therefore insufficient for efficiently exploring this design space. As the number of design variables and their interdependencies continue to increase, rational and predictive engineering approaches become increasingly important. Recent advances in artificial intelligence and machine learning enable the prediction of nucleic acid structures, optimization of functional sequences, identification of effective modification patterns, and establishment of quantitative structure-function relationships.^8^ By integrating computational prediction with iterative experimental validation, AI-assisted design may support a design-build-test-learn framework for the rational design of nucleic acid nanodevices.

These molecular, structural, functional, and computational engineering strategies have expanded the biomedical applications of nucleic acid nanodevices. Improved stability and targeting facilitate *in vivo* delivery, whereas programmable molecular recognition supports biosensing and disease diagnosis. Stimulus-responsive and logic-controlled behaviors enable context-dependent drug release and precision therapy, while the integration of imaging and therapeutic modules facilitates theranostic applications. Nevertheless, further translation will require advances in scalable manufacturing, quality control, pharmacokinetic optimization, long-term biosafety evaluation, and precise regulation of device behavior in complex biological environments.

Overall, our review highlights chemical engineering as a systematic design strategy for nucleic acid nanodevices. Rather than simply optimizing individual molecular properties, chemical engineering provides a comprehensive framework that connects molecular composition, structural organization, biological interaction, and dynamic function. Continued integration of chemical biology, nucleic acid nanotechnology, materials science, and AI-assisted design may facilitate the development of nucleic acid nanodevices from empirically optimized constructs toward more predictable platforms for precision diagnosis and therapy.

## Acknowledgments

This work was supported by the National Natural Science Foundation of China (22407044).

## Declaration of interests

The authors declare no competing financial interests.
